# Flexible Membranes of MoS_2_/C Nanofibers by Electrospinning as Binder-Free Anodes for High-Performance Sodium-Ion Batteries

**DOI:** 10.1038/srep09254

**Published:** 2015-03-24

**Authors:** Xiaoqin Xiong, Wei Luo, Xianluo Hu, Chaoji Chen, Long Qie, Dongfang Hou, Yunhui Huang

**Affiliations:** 1 State Key Laboratory of Materials Processing and Die & Mould Technology, School of Materials Science and Engineering, Huazhong University of Science and Technology, Wuhan 430074, P. R. China

## Abstract

A flexible membrane consisting of MoS_2_/carbon nanofibers has been fabricated by a simple electrospinning method. MoS_2_ nanosheets are uniformly encapsulated in the inter-connected carbon nanofibers with diameters of ~150 nm. When evaluated as a binder-free electrode for sodium-ion batteries, the as-obtained electrode demonstrates high performances, including high reversible capacity of 381.7 mA h g^−1^ at 100 mA g^−1^ and superior rate capability (283.3, 246.5 and 186.3 mA h g^−1^ at 0.5, 1 and 2 A g^−1^, respectively). Most importantly, the binder-free electrode made of MoS_2_ and carbon nanofibers can still deliver a charge capacity of 283.9 mA h g^−1^ after 600 cycles at a current density of 100 m A g^−1^, indicating a very promising anode for long-life SIBs.

Lithium-ion batteries (LIBs) have been the main power source for portable electronic devices and now are considered as the most promising technology for applications in electric vehicles (EVs) and green energy storage, because of their high energy density and long cycle life^1^,^2^. However, the rarity and uneven distribution of lithium resources may cause a potentially higher price of LIBs, which could significantly limit the further applications of LIBs in large-scale energy storage^3^. Compared to LIBs, sodium-ion batteries (SIBs) exhibit a similar electrochemical property but potentially a much lower cost due to the abundance and wide availability of sodium resources, which indicates a great promise for the large-scale energy storage^4^,^5^. Therefore, great efforts have been shifted to the development of SIBs in the last few years^4^,^5^.

To achieve the success of SIBs, the successful development of suitable electrode materials is crucial. Although the well-developed LIB technology provides a great guidance for SIBs, it is still a challenge to explore appropriate host materials with sufficiently large interstitial space to accommodate Na^+^ ions since the ionic size of Na^+^ ions is ca. 55% larger than that of Li^+^ ions^6^. In particular, graphite, the commercial anode for LIBs, only shows a very low capacity for SIBs because Na hardly forms staged intercalation compounds with graphite^7^. It is very critical to discover a high-performance anode for SIBs. To date, several types of SIB anodes have been studied, including (i) carbon-based materials^8^,^9^,^10^,^11^, (ii) alloys^12^,^13^, (iii) metal oxides^14^,^15^, (iv) metal chalcogenides^16^,^17^,^18^,^19^, etc. Among them, hard carbon has attracted significant attention due to its relatively high capacity and low cost. However, hard carbon often suffers from a poor rate capability and a fast capacity fading, which restricts its applications for high-power and long-life SIBs. Compared to hard carbon, theoretically, alloys and metal oxides could deliver higher capacities. Unfortunately, the alloy and metal oxide anodes usually experience a lower first-cycle Coulombic efficiency and a short lifetime due to the large volumetric change upon electrochemical discharge/charge cycling^12^,^20^,^21^. On the other hand, metal chalcogenides exhibit great performance as anodes for SIBs. Kitajouet *et al*. demonstrated that four Na^+^ ions could be inserted into the inter-layer space of FeS_2_ and thus delivered a high capacity of 758 mA h g^−1^ at 0.2 mA cm^−1^ 17. More recently, Qu *et al*. reported that SnS_2_/reduced graphene oxide composite as SIB anode materials delivered an initial capacity of 630 mA h g^−1^ and an unvarying capacity of 500 mA h g^−1^ even after 400 cycles^19^. Therefore, it is highly desirable to develop other metal chalcogenides as anodes for SIBs.

During the past decades, molybdenum disulfide (MoS_2_) has been extensively investigated in a variety of fields, such as, catalysis^22^, solid lubricants^23^, hydrogen storage^24^, and supercapacitors^25^,^26^, due to its unique physical/chemical properties. Recently, when evaluated as an anode for LIBs, MoS_2_ also shows a very high theoretical capacity (~670 mA h g^−1^, based on 4 mol of Li^+^ insertion), great rate capability and good cyclability^27^,^28^,^29^. Inspired by this, early studies have demonstrated that MoS_2_ can also be a promising anode for SIBs considering its high capacity. However, it is reported that the MoS_2_ electrode exhibits a fast capacity fading and inferior rate capability because of its large volume change and poor electrical/ionic conductivity between two adjacent S-Mo-S sheets^30^,^31^,^32^. To solve these issues, many efforts have been devoted to fabricating MoS_2_/C hybrid nanostructures, which can effectively enhance the electrical conductivity of MoS_2_ electrodes and improve the structure stability as well^33^,^34^,^35^,^36^. These early studies have made a significant progress. However, the low first-cycle Columbic efficiency and short lifetime of MoS_2_ electrodes still need to be settled. Herein, we report on the large-scale flexible membrane of MoS_2_/carbon nanofibers (MoS_2_-CNFs) by electrospinning. When evaluated as the binder-free anode for SIBs, the as-formed MoS_2_-CNFs membrane shows high capacity, good rate capability and stable cycling performance.

## Results and Discussion

**Figure 1:**
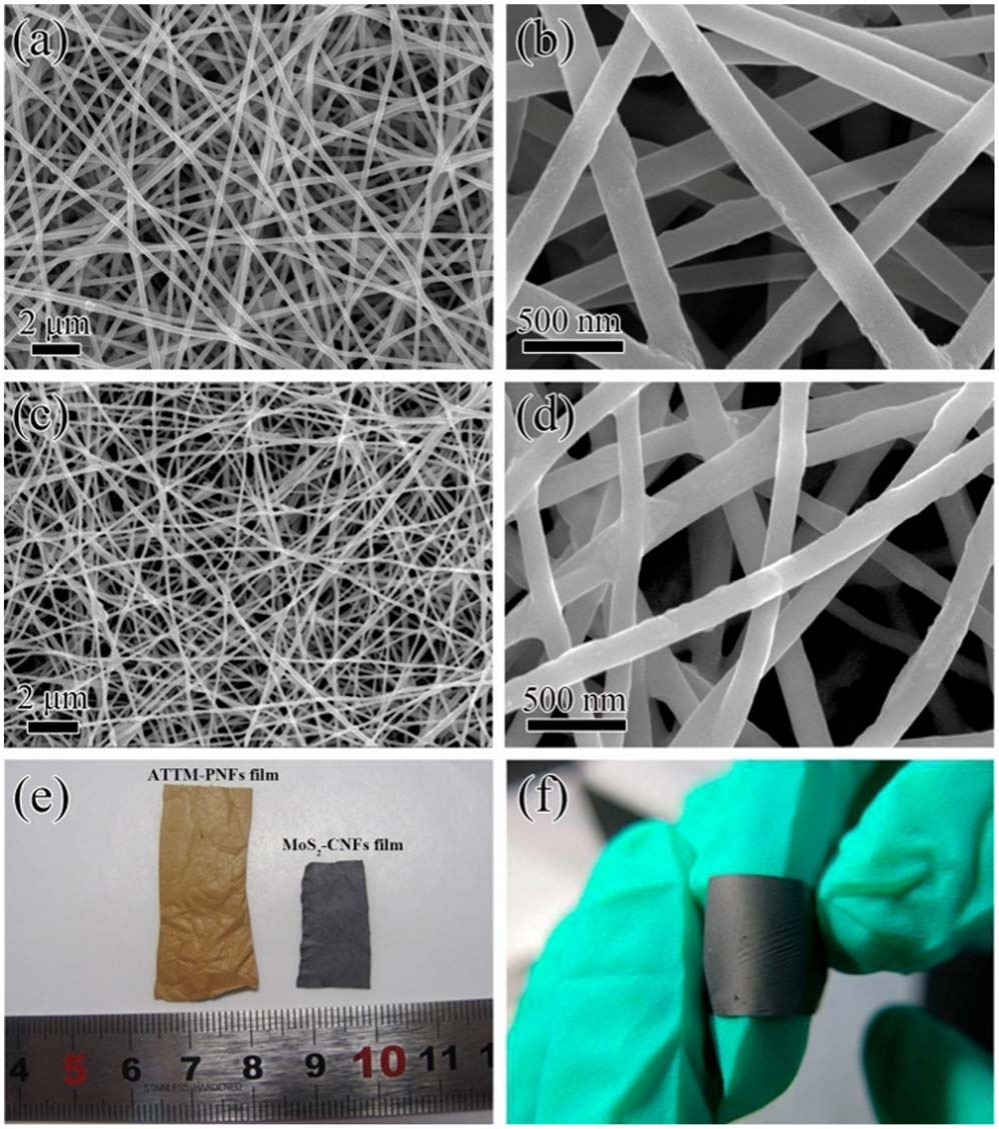
SEM Morphologies of the as-spun nanofibers and the fibers after thermal treatment in Ar/H_2_ at 400°C for 2 h, then, 800°C for 1 h. (a, b) FESEM images of the as-spun ATTM-PAN film; (c, d) FESEM images of the resulting MoS_2_-CNFs film; (e) Digital photo for the ATTM-PAN and MoS_2_-CNFs films; (f) Digital photo shows the flexible property of the as-obtained MoS_2_-CNFs film.

**Figure 2:**
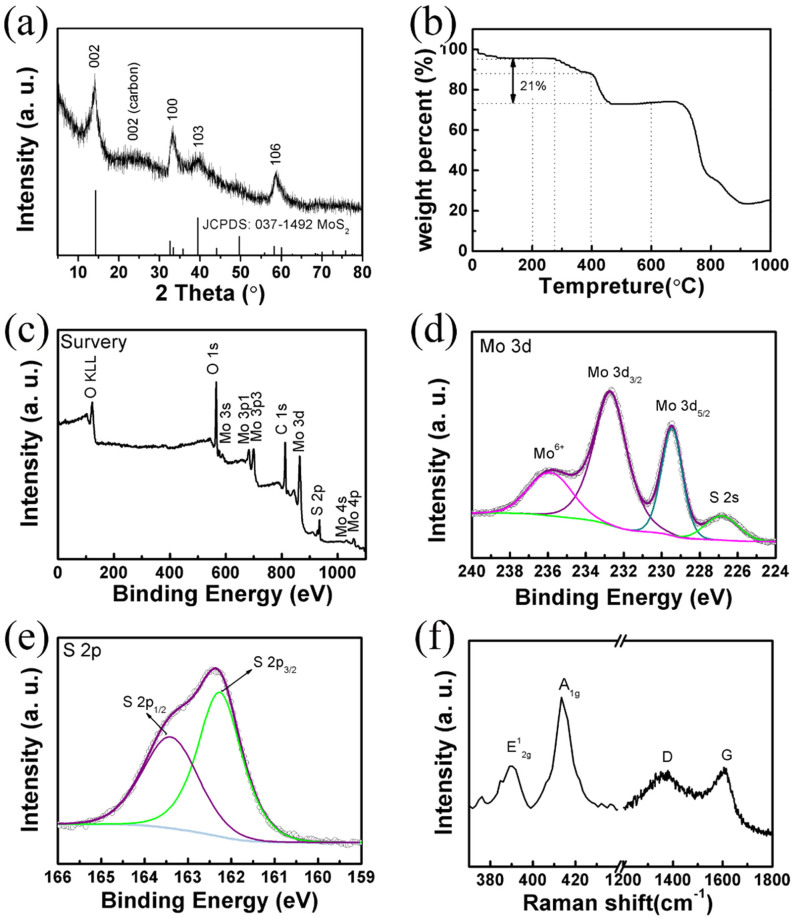
Structural information of MoS_2_-CNFs *via* XRD, TG, XPS and Raman. (a) XRD pattern; (b) TGA curve of the MoS_2_-CNFs film; (c) XPS survey spectrum of the as-prepared MoS_2_-CNFs. High resolution XPS spectra of (d) Mo 3d and (e) S 2p; (f) Raman spectrum of the as-prepared MoS_2_-CNFs.

The morphologies of the as-prepared products are studied by field-emission scanning electron microscopy (FESEM). As expected, the as-spun ATTM-PAN nanofibers are comprised of a large amount of interconnected one dimensional (1D) nanofibers (Fig. 1a). At a higher magnification (Fig. 1b), it can be found that these 1D nanofibers have a smooth surface with an average diameter of ~200 nm. After thermal treatment, the 1D structure and smooth surface is well maintained for MoS_2_-CNFs while the diameter shrinks to ~150 nm (Fig. 1c and 1d). More importantly, the MoS_2_-CNFs films exhibit excellent membrane flexibility that it can be easily cut into disks as free-standing, binder-free electrodes (Fig. 1e and 1f). The energy-dispersive X-ray (EDX) spectrum is further collected to determine the chemical compositions. As indicated in Fig. S1, the peaks of Mo, S, C and O elements clearly appear in the MoS_2_-CNFs product. The crystallinity and phase information of the MoS_2_-CNFs film is examined by X-ray diffraction (XRD), as shown in Fig. 2a. The diffraction peaks of the MoS_2_-CNFs can be indexed to the hexagonal phase of MoS_2_ (JCPDS No.37-1492). The diffraction peaks at 14.05°, 33.28°, 39.00° and 58.50° are assigned to the (002), (100), (103) and (110) planes, respectively. Interestingly, the diffraction peak of 002 at 14.05° corresponds an interlayer distance of 0.64 nm, which is slightly larger than 0.62 nm for MoS_2_ in previous reports^37^. This increased interlayer distance suggests that the layered MoS_2_ grows well along the C-axis during annealing. Moreover, an additional broad diffraction peak at ~25° should relate to the (002) plane of the carbon in the MoS_2_-CNFs. In order to determine the content of C in the MoS_2_-CNFs composite, thermogravimetric analysis (TGA) measurement is conducted. As shown in Fig. 2b, there are five steps of weight loss in the TGA curve. The weight loss of first 4.5% before 200°C belongs to the water evaporation. The oxidation of MoS_2_ to MoO_3_ occurs at 280–405°C. The slopping part between 405°C and 485°C is assigned to the combustion of carbon. The weight loss at the temperature higher than 680°C is due to the evaporation of MoO_3_ in air atmosphere. The content of MoS_2_ in MoS_2_-CNFs is calculated to be approximately 83.2% (Fig. S2).

To further study the chemical composition and the surface electronic states of MoS_2_-CNFs, X-ray photoelectron spectroscopy (XPS) analysis was carried out. The survey XPS spectrum (Fig. 2c) indicates the presence of Mo, S, C and O elements in the MoS_2_-CNFs film, which is consistent with the EDX results. The high-resolution XPS spectra of Mo 3d and S 2p are shown in Fig. 2d and 2e, respectively. The peaks at 232.8 and 229.5 eV are related to the Mo 3d_3/2_ and Mo 3d_5/2_, corresponding to Mo^4+^ in MoS_2_-CNFs^26^,^37^. The presence of another XPS peak at ~236.0 eV is indexed to Mo^6+^ 3d_5/2_ of MoO_3_, which may be resulted from the surface oxidation at the MoS_2_-CNFs in air^38^,^39^. Moreover, the XPS peaks at 162.4 and 163.5 eV in S 2p spectra are characteristic of the S^2−^ of MoS_2_ (Fig. 2e). The typical Raman spectrum for the MoS_2_-CNFs (Fig. 2f) displays the characteristic D- and G-bands of carbon at 1350 and 1580 cm^−1^, respectively. Also, the peaks at 390.5 and 413.5 cm^−1^ correspond to the E^1^_2g_ and A^1^_g_ modes of MoS_2_. It has been reported that the energy difference between the two Raman peaks can be used to identify the number of layers in few-layer MoS_2_ crystals^40^,^41^. In this study, this energy difference is 23 cm^−1^, confirming that MoS_2_ is single-layer or few-layer and embedded in/on the carbon nanofibers in the MoS_2_-CNFs products.

**Figure 3:**
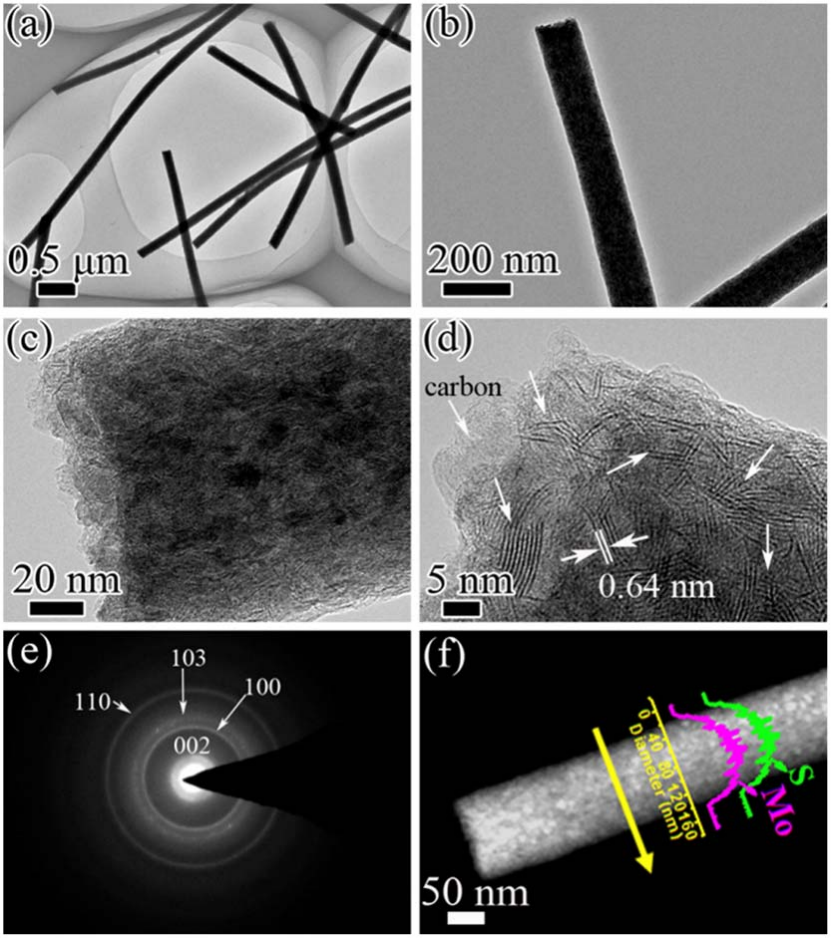
TEM Morphologies of the as-prepared MoS_2_-CNFs nanofibers. (a, b) TEM images and (c, d) HRTEM images of MoS_2_-CNFs, The arrows in (d) indicate the separated layers with a thickness ~0.64 nm; (e) SAED pattern; (f) dark-field TEM image, and the corresponding EDS line scan profiles for Mo and S along the line of MoS_2_-CNFs.

The low-magnification transmission electron microscopy (TEM) images (Fig. 3a and 3b) confirm that MoS_2_-CNFs consist of interconnected nanofibers with a diameter of ~150 nm. The high-resolution TEM (HRTEM) images demonstrate that MoS_2_ nanosheets with layered structure are uniformly distributed on the surface and/or embedded in the CNFs framework (Fig. 3c and 3d). It should be noted that most of the building blocks in our sample are single or several layers^42^. Moreover, the arrows marked in Fig. 3d exhibit that the interlayer distance between the layers of MoS_2_ is 0.64 nm. It agrees well with the XRD results that the layered MoS_2_ grows well along the C-axis during annealing. The selected area electron diffraction (SEAD) pattern in Fig. 3e can be indexed to the hexagonal MoS_2_ phase where these diffraction rings can be indexed to the (002), (100), (103) and (110) planes, respectively. Fig. 3f shows the EDX line-scan analysis, which also confirms the homogeneous distribution of Mo and S along the line crossing the nanofiber.

**Figure 4:**
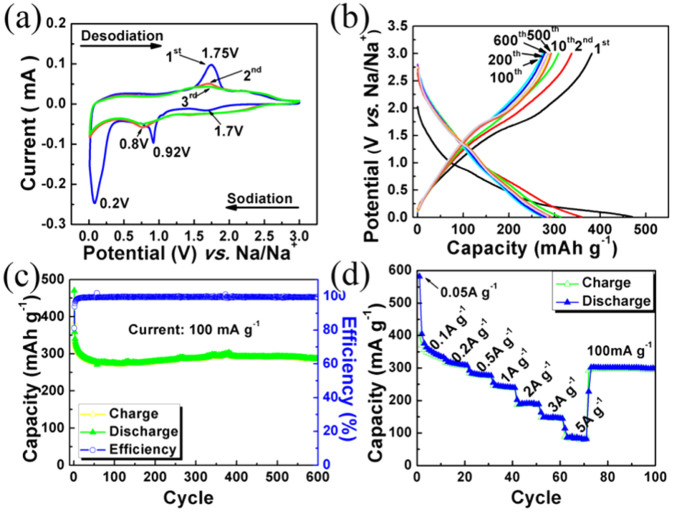
Electrochemical performances. (a) Initial three cycles of CV curves for the self-standing MoS_2_-CNFs electrode at a scan rate of 0.2 mV s^−1^; (b) Galvanostatic discharge/charge profiles of the MoS_2_-CNFs electrode at a current density of 100 mA g^−1^ within the potential range 0.01–3.0 V *vs*. Na^+^/Na; (c) Cycling performance and corresponding coulombic efficiency of the MoS_2_-CNFs electrode at 100 mA g^−1^; (d) Rate capacity of the MoS_2_-CNFs at different current densities in the potential range of 0.01–3.0 V.

Owing to its great self-standing and flexible properties, the MoS_2_-CNFs film is cut into a disk as a binder-free electrode. Fig. 4a displays a typical CV curve of the MoS_2_-CNFs electrode in the potential window of 0.01–3.0 V. Three reduction peaks in the first sodiation process were observed, which are situated at 1.7, 0.92 and 0.2 V, respectively. The reduction peak at 1.7 V is attributed to the insertion of Na^+^ ions into MoS_2_, forming Na*_x_*MoS_2_^43^,^44^. The following reduction peak at 0.92 V is related to the further insertion of Na^+^ ions in combination with the formation of a layer of solid electrolyte interphase (SEI). The sharp peak at 0.2 V indicates the conversion reaction (Na*_x_*MoS_2_ + Na^+^→ Mo + Na*_x_*S)^44^,^45^,^46^. In the following oxidation curve, the peak near 1.75 V corresponds to the desodiation. Interestingly, the CV curves for the 2nd and 3rd cycle are nearly overlapped, indicating a good reversibility and predominance of the storage reactions.

The galvanostatic discharge/charge behavior of MoS_2_-CNFs was measured at a current density of 100 mA g^−1^ over a potential range of 0.01–3.0 V *vs.* Na^+^/Na. As shown in Fig. 4b, the first discharge process (insertion of Na^+^ ions into MoS_2_-CNFs) shows the correlative plateau regions that are identified to the CV profiles in the first discharge process at around 0.8 and 0.2 V, suggesting the insertion and conversion process of MoS_2_. The initial discharge and charge capacities are 470.2 and 381.7 mA h g^−1^, corresponding to a coulombic efficiency (CE) of 81.2%. Fig. 4c exhibits the cycling performance of MoS_2_-CNFs electrodes at a current density of 100 mA g^−1^. The initial charge capacity of the MoS_2_-CNFs are 381.7 mA h g^−1^. Finally, the electrodes of MoS_2_-CNFs maintain a capacity of 283.9 mA h g^−1^ of charge capacity after 600 cycles, and the capacity retention rate is 74.8%. For comparison, the electrochemical properties of the bulk MoS_2_ and P-CNFs are show in Fig. S5. The rate capacities display highly reversible storage properties as well as the cycling performance. As shown in Fig. 4d, the hybrid MoS_2_-CNFs flexible film delivers reversible capacities of 400.6, 369.7, 316.9, 283.3, 246.5, 186.3,148 and 89 mA h g^−1^ at current densities of 0.05, 0.1, 0.2, 0.5, 1, 2, 3 and 5 A g^−1^, respectively. Importantly, when the current density resumed to 0.1 A g^−1^ after cycling at different rates, a capacity of 292 mA h g^−1^ was achieved. This further confirms the stable structure of the nanofiber-based hybrid film and excellent reversibility. In our case, the few layered MoS_2_ embedded in the amorphous carbon fibers could shorten sodium ion and electron diffusion distances. In order to explore the structural stability of the electrode, we further investigated the microstructure after 500 continuous discharge/charge cycles (Fig. S6). It was found that some of the fibers are nanosized, but most of the fibers are still maintained. Also, the rate capacity of MoS_2_-CNFs composites is much improved in comparison to bulk MoS_2_ and P-CNFs (Fig. S5b and S5d).

**Figure 5:**
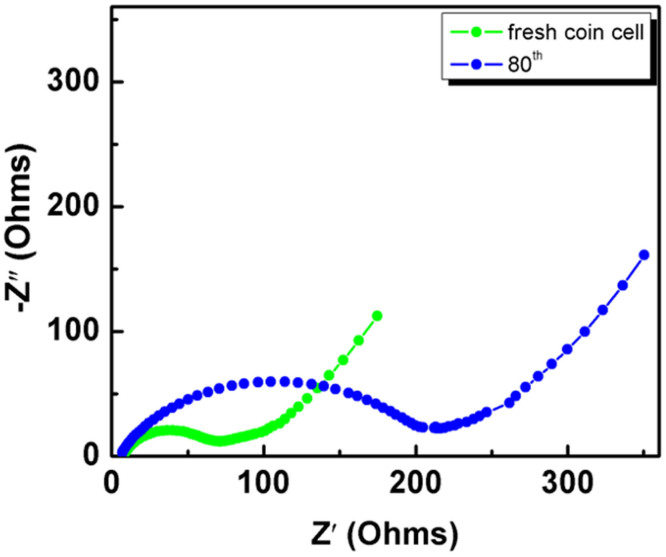
Nyquist plots of the MoS_2_-CNFs free-standing electrode.

The electrochemical impedance spectra of the MoS_2_-CNFs electrode are investigated after different discharge/charge cycles over the frequency range from 100 kHz to 0.1 Hz (Fig. 5). After continuous discharge/charge cycling, the Nyquist plots of the MoS_2_-CNFs electrode are similar, displaying a depressed semicircle in the high-middle frequency region and an oblique straight line in the low frequency region. The diameter of the semicircle of the fresh cell is very small, suggesting that the MoS_2_-CNFs hybrid electrodes possess low contact and charge-transfer impedances. Only a slight increase was found in semicircle diameters even after 80 discharge/charge cycles, indicating the good stability of the as-prepared electrodes. It is well known that electrode pulverization and poor cycling stability are caused by the vigorous volume expansion and particle aggregation associated with Na^+^ insertion and extraction processes. The electrochemical impedance spectroscopy (EIS) and morphology studies confirm that the homogeneous distribution of few-layered MoS_2_ nanosheets in the hybrid nanofibers as well as its free-standing structural nature contribute to the excellent cycling stability.

In summary, a simple and scalable electrospinning method has been successfully developed to fabricate flexible MoS_2_-CNFs membranes as binder-free anodes for SIBs. The as-prepared MoS_2_-CNFs membranes with homogeneous few-layered MoS_2_ distributed in the carbon nanofibers exhibit high capacity, superior rate capability (283.3, 246.5 and 186.3 mA h g^−1^ at 0.5, 1 and 2 A g^−1^, respectively), and outstanding cyclability. The present strategy may be extended to fabricate other flexible nanocomposite membranes serving as high-performance binder-free electrodes for future SIB applications.

## Methods

### Synthesis of MoS_2_-CNFs

MoS_2_/C nanofibers were prepared by a simple electrospinnig route followed by a post-treatment process^47^,^48^. Briefly, a mixed solution for electrospinning was prepared from polyacrylonitrile (PAN, Aladdin Chemical Co., Ltd.), ammonium tetrathiomolybdate [ATTM, (NH_4_)_2_MoS_4_, 99.99%, Alfa], and N, N-dimethylformamide (DMF, C_3_H_7_NO, sinopharm). All chemicals were used as received without further purification. In a typical procedure, 2.4 g of ATTM was dissolved in DMF (10 ml) by stirring overnight. Then, 0.8 g of PAN was added into the above solution. The mixture of ATTM-PAN was further stirred overnight to get a dark-red homogeneous solution. Then, the precursor solution was transferred into a plastic syringe equipped with a 20-gauge stainless steel needle. The feeding rate was 0.3 mL h^−1^ monitored by a syringe pump. The metallic needle clamped with an electrode was connected to a high-voltage power supply, and a collector of aluminum foil as a grounded counter electrode was 12 cm away from the tip of the needle. As a high voltage of 15 kV was applied, the ATTM-PAN nanofibers were formed. The collected as-electrospun fibers were stabilized at 400°C for 2 h and carbonized at 800°C for 1 h in Ar (95 vol%)/H_2_ (5 vol%) to achieve MoS_2_-CNFs. In a control experiment, pure carbon fibers (P-CNFs) were prepared by a similar procedure without adding ATTM and its precursor was named PAN-NFs, and bulk MoS_2_ was commercial product and purchased from aladdin.

### Electrochemical measurements

Electrochemical experiments were performed using two-electrode CR2032 coin cells. MoS_2_-CNFs films were directly cut into disks as the working electrodes. For P-CNFs, the working electrodes were fabricated by coating a slurry containing 70 wt% P-CNFs, 20 wt% acetylene black (Super-P) and 10 wt% polyvinylidene fluoride (PVDF) binder onto a copper foil. A sodium pellet was used as the counter electrode, a glass fiber membrane as a separator and the solution of 1.0 M NaClO_4_ in ethylene carbon (EC)/propylene carbonate (PC) (*v*/*v* = 2:1) as the electrolyte. Galvanostatic charge-discharge was performed on a multichannel battery testing system (Land, China) and cyclic voltammetry (CV) was measured by CHI600D (shanghai, China) in a voltage range of 3–0.01 V *vs*. Na^+^/Na at a scanning rate of 0.2 mV s^−1^ at room temperature.

### Other Characterizations

Powder X-ray diffraction (XRD) patterns were collected by a PANalytical Multi-Purpose Diffractometer using high-intensity Cu K_α1_ irradiation (*λ* = 1.5406 Å). The morphology and composition of the products were characterized using field-emission scanning electron microscopy (FESEM, FEI Sirion 200) coupled with an energy-dispersive X-ray (EDX) spectrometer. The transmission electron microscopy (TEM) images were obtained with Tecnai G2 F30 (FEI, Holland) transmission electron microscope. X-ray photoelectron spectroscopy (XPS) measurements were carried out on a VG MultiLab 2000 system with a monochromatic Al K X-ray source (Thermo VG Scientific). Raman spectra were collected using a Bruker VERTEX 70. The carbon contents were determined by thermogravimetric analysis (TG, PerkinElmer) performed under air atmosphere at a heating rate of 10°C min^−1^ from room temperature to 1000°C.

## Supplementary Material

Supplementary Information Supporting Information
