# Supplementary material for: Flexible Membranes of MoS2/C Nanofibers by Electrospinning as Binder-Free Anodes for High-Performance Sodium-Ion Batteries
Source: Sci Rep. 2015 Mar 24;5:9254. doi: 10.1038/srep09254 (PMC5380159; doi:10.1038/srep09254)
Supplement: Supplementary Information — Supporting Information [file srep09254-s1.doc]

**SUPPLEMENTARY INFORMATION FOR**

Flexible Membranes of MoS_2_/C Nanofibers by Electrospinning as Binder-Free Anodes for High-Performance Sodium-Ion Batteries

Xiaoqin Xiong, Wei Luo, Xianluo Hu,* Chaoji Chen, Long Qie, Dongfang Hou &Yunhui Huang*

State Key Laboratory of Materials Processing and Die & Mould Technology, School of Materials Science and Engineering, Huazhong University of Science and Technology, Wuhan 430074, P. R. China.

Correspondence and requests for materials should be addressed to X.L.H. (huxl@mail.hust.edu.cn) or Y.H.H. ([huangyh@mail.hust.edu.cn](mailto:huangyh@mail.hust.edu.cn))


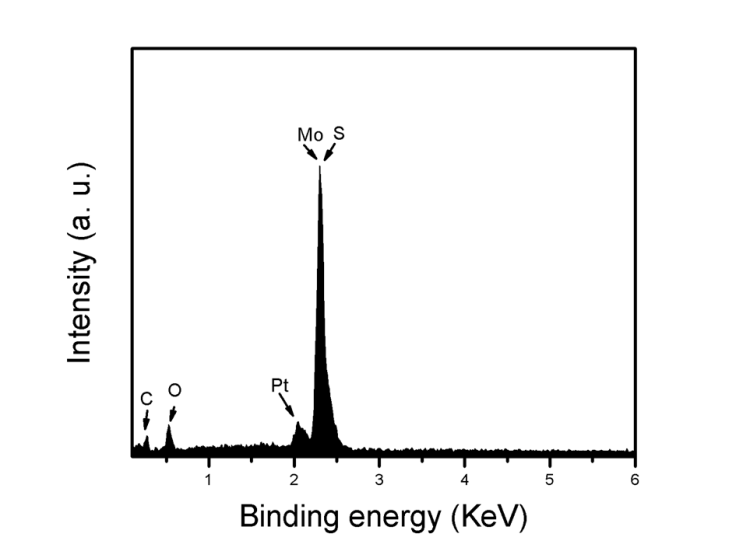


**Figure S1. EDX spectrum of the MoS_2_-CNFs film**


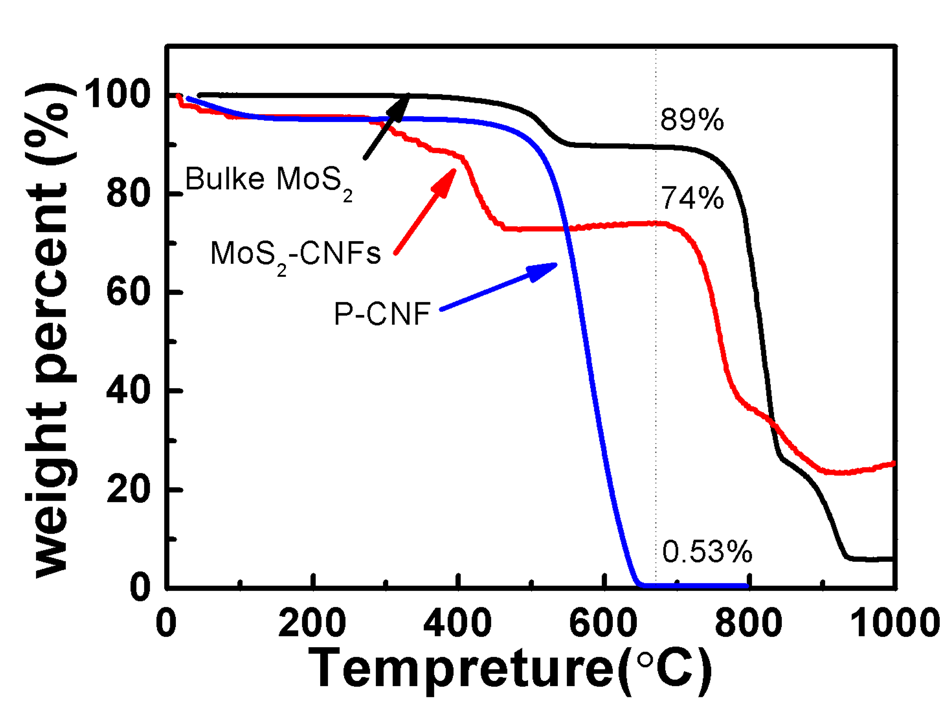


**Figure S2. TG analysis curves of bulk MoS_2_, P-CNFs and MoS_2_-CNFs under flowing air at a heating rate of 10 ºC min^-1^.** The residual weight percent of bulk MoS_2_, P-CNFs and MoS_2_-CNFs at 680 ºC is shown in the TG curve and used to calculate the content of MoS_2_ in the MoS_2_-CNFs based on the equation W_MoS2_*X* _MoS2_, + W_P-CNFs_ (1-*X*_MoS2_,) = W_MoS2-CNFs_, where *X*_MoS2_ is labled as the content of MoS_2_ in the MoS_2_-CNFs and W_MoS2_ W_P-CNFs_ W_MoS2-CNFs_ are signified as residual weight percent of bulk MoS_2_, P-CNFs and MoS_2_-CNFs at 680 ºC. By calculation, the content of MoS_2_ in the MoS_2_-CNFs is approximately 83.2%.


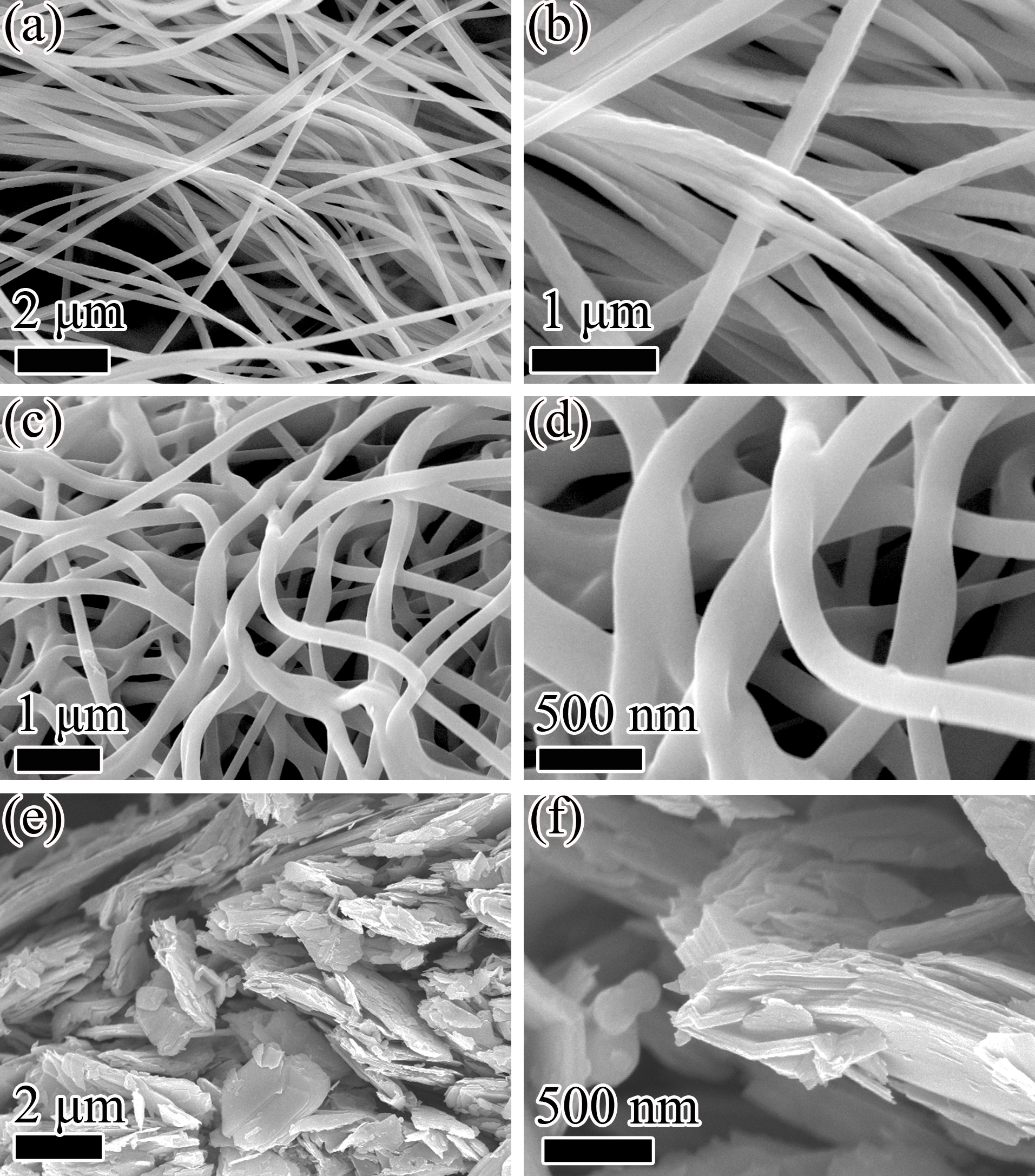


**Figure S3. FESEM images of the as-prepared materials.** (a, b) PAN-NFs, (c, d) P-CNFs and (e, f) bulk MoS_2_.


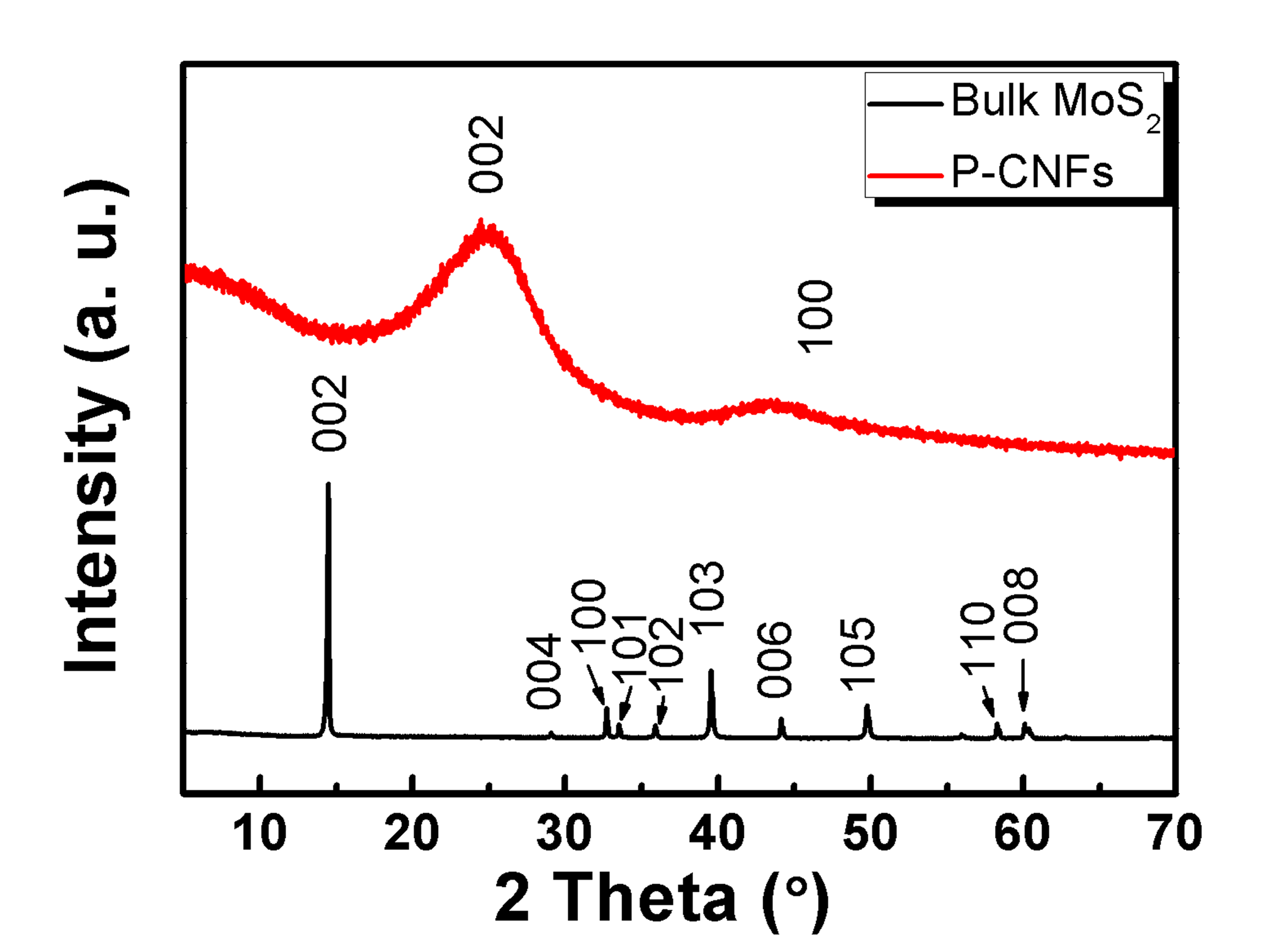


**Figure S4. XRD patterns of bulk MoS_2_ and P-CNFs.**


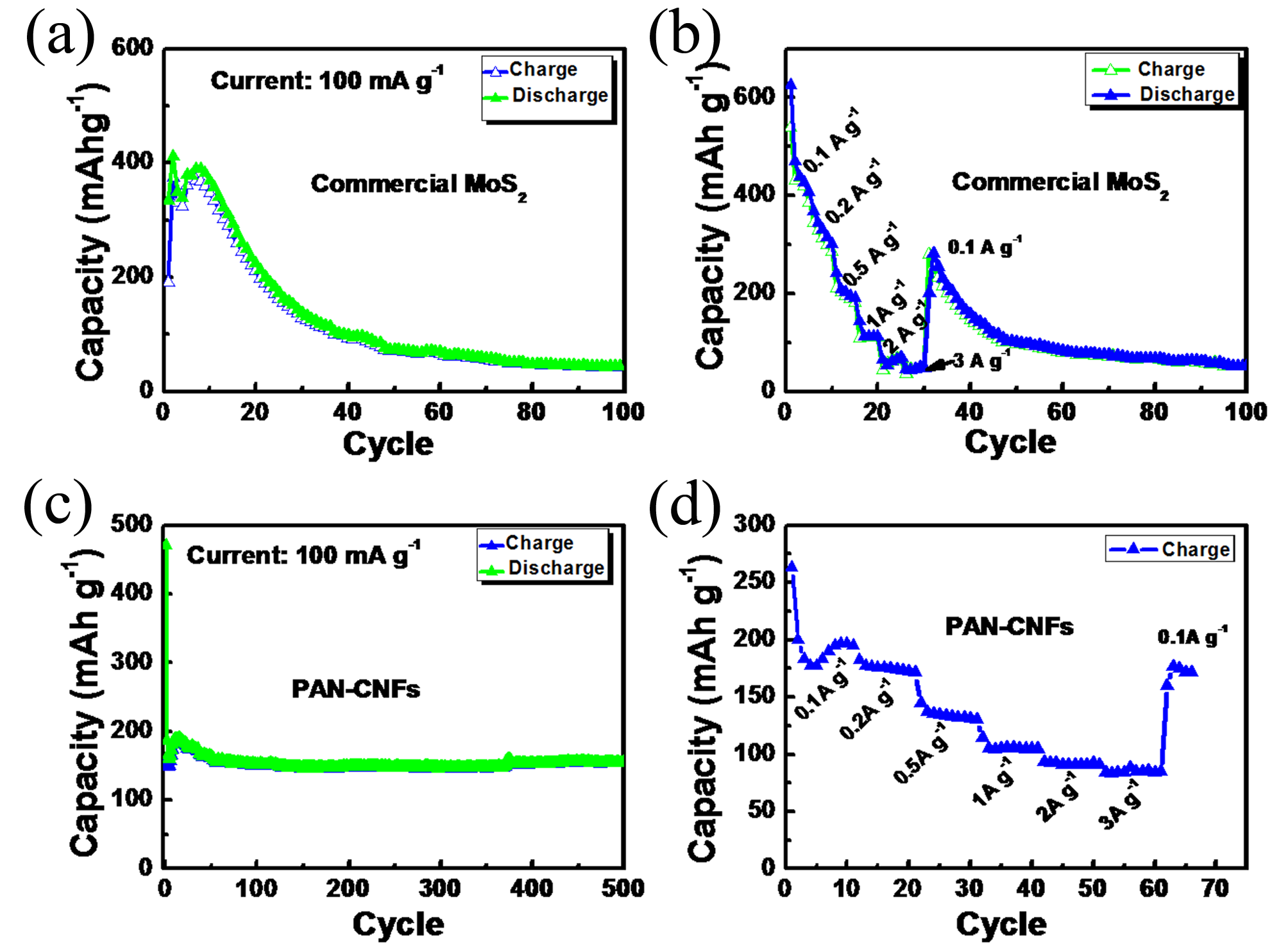


**Figure S5. Electrochemical performances of bulk MoS_2_ and P-CNFs.** (a, c) Cycling performance of bulk MoS_2_ and P-CNFs at a current density of 100 mA g^–1^ over a potential range 0.01–3.0V *vs*. Na/Na^+^. (b, d) Rate capacity of the bulk MoS_2_ and P-CNFs.


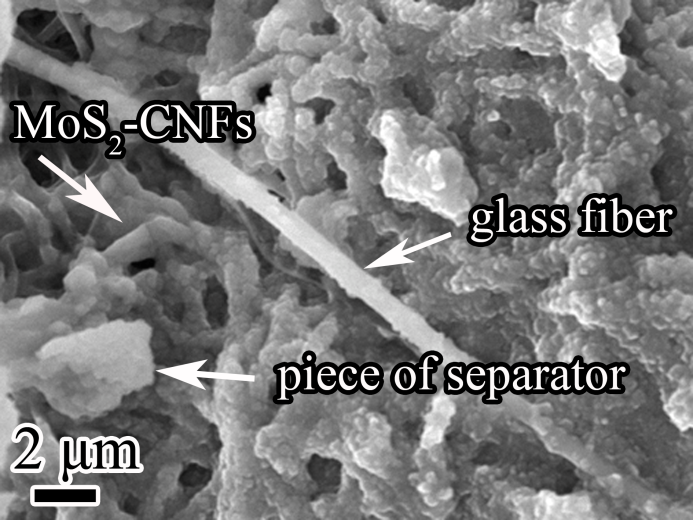


**Figure S6. SEM image of the electrode after continuous 500 discharge/charge cycles at a C-rate current and then back to 0.1 A g^–1^.**
